# Usage and impact of patient‐reported outcomes in epilepsy

**DOI:** 10.1002/brb3.3342

**Published:** 2023-11-23

**Authors:** Kristl Vonck, Arnaud Biraben, Magdalena Bosak, Poul Jørgen Jennum, Vasilios K Kimiskidis, Petr Marusic, James W. Mitchell, Lara N. Ferreira, Martina Ondrušová, Adrian Pana, Ulf Persson, Tim J. von Oertzen, Simona Lattanzi

**Affiliations:** ^1^ Department of Neurology, 4Brain Ghent University Hospital Gent Belgium; ^2^ Unité d’épileptologie, CHU Pontchaillou Rennes Rennes France; ^3^ Department of Neurology Medical College Jagiellonian University Krakow Poland; ^4^ Department of Clinical Neurophysiology, Danish Center for Sleep Medicine Rigshospitalet Denmark; ^5^ First Department of Neurology AHEPA University Hospital, Aristotle University of Thessaloniki Thessaloniki Greece; ^6^ Department of Neurology Second Faculty of Medicine and Motol University Hospital, Charles University Prague Czech Republic; ^7^ Institute of Systems, Molecular and Integrative Biology University of Liverpool Liverpool UK; ^8^ Universidade do Algarve—ESGHT Faro Portugal; ^9^ Centre for Health Studies and Research of the University of Coimbra (CEISUC), Centre for Innovative Biomedicine and Biotechnology (CIBB) Coimbra Portugal; ^10^ Research Centre for Tourism Sustainability and Well‐Being (CinTurs), Universidade do Algarve Faro Portugal; ^11^ Department of Epidemiology and Biostatistics, Pharm‐In, Ltd. Bratislava Slovakia; ^12^ Faculty of Public Health Slovak Medical University Bratislava Slovakia; ^13^ School of Public Health, Babes Bolyai University Cluj Napoca Center for Health Outcomes & Evaluation Cluj‐Napoca Romania; ^14^ The Swedish Institute for Health Economics Lund Sweden; ^15^ Center for Medicine of the Elderly, Kepler University Hospital Johannes Kepler University Linz Austria; ^16^ Neurological Clinic, Department of Experimental and Clinical Medicine Marche Polytechnic University Ancona Italy

**Keywords:** economics, epilepsy, patient‐reported outcomes, seizure

## Abstract

**Background:**

The use of patient‐reported outcomes (PRO) in clinical practice is gaining increasing attention. This study aimed to provide a critical assessment of the current state‐of‐the‐art and beliefs about the use of PRO in the management of people with epilepsy across some European countries.

**Methods:**

Structured interviews were conducted with European experts to collect insights about (I) the personal experience with PRO; (II) the value and impact of PRO in the decision‐making process at the national level; and (III) the interest for and use of PRO by national health authorities.

**Results:**

Nine neurologists (Austria, Belgium, Czechia, Denmark, France, Greece, Italy, Poland, and United Kingdom), three health economists (Portugal, Romania, and Sweden), and one epidemiologist (Slovakia) participated. They all stated that PRO are collected at their own countries in the context of clinical trials and/or specific projects. During everyday clinical practice, PRO are collected routinely/almost routinely in Austria and Sweden and only at the discretion of the treating physicians in Czechia, Denmark, France, Greece, and Portugal. There was complete consensus about the favorable impact that the PRO can have in terms of clinical outcomes, healthcare resources utilization, and general patient satisfaction. Only participants from Portugal and Sweden answered that the PRO are perceived as very important by the National Health Authorities of their respective countries.

**Conclusions:**

Differences exist in attitudes and perspectives about PRO in epilepsy across Europe. An active plan is warranted to harmonize the measurement of PRO and ensure they can be relevant to people with epilepsy and health services.

## 1 INTRODUCTION

Patient‐reported outcomes (PRO) are any report of the status of a patient's health condition that comes directly from the patient, without interpretation of the patient's response by anyone else (Food & Drug Administration, U.S. Department of Health & Human Services: Guidance for Industry, 2009). The use of PRO measures in clinical practice is gaining increasing attention. Potential benefits include the improvement and facilitation of patient‐clinician communication, a better assessment of functional or mental health domains of patients, a more accurate monitoring of the effects of treatment on the health of patients, a more informed clinical decision‐making, and support to patient self‐management (Marshall et al., 2006; Santana & Feeny, 2014; Valderas et al., 2008). The integration of PRO measures into routine clinical practice may also offer the opportunity to allocate healthcare resources in a more rational way on the basis of the individualized needs and facilitate delivering “value‐based” and equitable health care (Catalyst, 2017; Schougaard et al., 2018).

Epilepsy represents one of the most common noncommunicable brain disorders and affects approximately 70 million people worldwide (Ngugi et al., 2010; Ngugi et al., 2011). This disease is characterized by recurrent seizures, which are unpredictable and have significant negative repercussions, increasing the risk of injury, hospitalization, and mortality (Lattanzi et al., 2022). The burden of epilepsy, however, goes beyond the consequences of seizures alone, and extends into psychological and social aspects of daily life (Christensen et al., 2023; Jennum et al., 2016; Jennum et al., 2017; Kerr, 2012).

The care of persons with epilepsy, therefore, requires a holistic and tailored approach that considers their individual needs. Importantly, several of the different aspects of health affected by epilepsy can be reported accurately only by the patients themselves and PRO measures can represent a tool to explore and evaluate these issues. Although the systematic collection of PRO has been shown to be feasible in the clinical setting (Delgado‐García et al., 2021; Moura et al., 2016), several barriers still exist for their widespread use in everyday practice (Boyce et al., 2014; Lohr & Zebrack, 2009; Porter et al., 2016; Wright, 2000). In addition, there exists a great heterogeneity in the way epilepsy is managed across different countries (Meyer et al., 2010).

This study aimed to provide a critical assessment of the current state‐of‐the‐art and beliefs about the use of PROs in the management of people with epilepsy across some European countries and offer insights toward current unmet needs and future perspectives on the topic.

## 2 METHODS

### 2.1 Participants and procedures

Between November and December 2022, structured interviews were conducted with European experts working at tertiary, academic centers to collect insights about PRO in epilepsy. The participants were selected based on their experience in the fields of neurology, health economy, and epidemiology, the country of origin (no more than one participant per country), and previous collaborations. Due to the preliminary and explorative nature of the study, the selection of the expert partners in this project was not randomly assigned nor based on a systematic approach.

The interview aimed to collect insights on the experience and point of view about PRO in epilepsy. In particular, the interview included questions about (I) the personal experience with PRO; (II) the value and impact of PRO in the decision‐making process at the national level; and (III) the interest for and use of PRO by national health authorities (Appendix). The interviews had an average duration of 25 min, were conducted in English via video call, and recorded.

### 2.2 Statistical analysis

We descriptively summarized the responses from the participants. Values were presented as number of responders for each question.

## 3 RESULTS

The respondents to the interview were eight neurologists with special interest in epilepsy (from Austria, Belgium, Czechia, Denmark, France, Greece, Italy, and Poland), one neurologist in training with a research interest in outcome measurement (from the United Kingdom), three health economists (from Portugal, Romania, and Sweden), and one epidemiologist (from Slovakia). A summary of the responses to the interview is provided in Tables 1, 2, 3.

**TABLE 1:** Answers of participants to the structured interview: Part I.

Part I. patient‐reported outcomes (PROs): collection during patient access, treatment, and follow‐up	
**Are PROs collected within your center?** Yes	13/13
**Which professional figure is in charge of the collection and analysis of PRO in your center?**	
Clinician	13/13
Nurse	4/13
Other	3/13
**When are PRO collected?**	
In clinical trials and/or specific projects	13/13
Routinely/almost routinely during everyday clinical practice	2/13
At the discretion of the treating physicians during everyday clinical practice	5/13
**If PRO are collected during everyday clinical practice, are they collected in the patient access phase, investigating quality of life, impact of the illness on the productivity, concerns, or expectations from the treatment?**	
Yes	7/7
**If PRO are collected during everyday clinical practice, are they collected during the treatment phase, as parameters to define the impact of the therapy on quality of life and the patient's functional status?**	
Yes	7/7
**If PRO are collected during everyday clinical practice, does the collection continue during the follow‐up phase?**	
Yes	7/7
**If PRO are collected during everyday clinical practice, how are PRO data used in your center?**	
To evaluate the efficacy and/or safety of the treatment	7/7
To inform decisions on the treatment	6/7
To inform decisions on the general management (e.g., need for a visit or other resources)	7/7
To help measure health care resources utilization and improve efficiency	5/7
**If PRO are collected during everyday clinical practice, which tools does your center use to collect them?**	
Paper‐based questionnaires	6/7
Electronic questionnaires	6/7
**Within your center, are PRO considered and included in the drug evaluation and selection process?**	
Yes	5/13
**If you could, would you dedicate more time/space to PRO collection?**	
Yes	9/13
**Is PRO collection considered a priority at your center?**	
Yes	5/13

*Note*: Number of participants to the interview who gave any answers out of the total number of participants are provided.

Abbreviation: PRO = patient‐reported outcomes.

**TABLE 2:** Answers of participants to the structured interview: Part II.

Part II. Value and impact of patient‐reported outcomes (PROs) in the decision‐making process	
**Do you think that the use of PRO and the evidence obtained from them, can offer a concrete advantage in terms of clinical outcomes, rational utilization of healthcare resources, and general patient satisfaction?** Yes	13/13
**Do you think that PRO collection and evaluation are useful to measure the success of a pharmacological treatment?** Yes	13/13
**Do you think that PRO collection and evaluation are useful to improve efficiency in patients’ management?** Yes	13/13
**Do you think that PRO can be included among the indicators of the value of a drug?** Yes	13/13
**Do you believe that PRO are useful indicators in orienting the choice toward one drug over another?** Yes	13/13
**Do you believe that PRO should be used to support label claims (product indications)?** Yes	10/13

*Note*: Number of participants to the interview who gave any answers out of the total number of participants are provided.

Abbreviation: PRO = patient‐reported outcomes.

**TABLE 3:** Answers of participants to the structured interview: Part III.

Part III. Interest for and use of patient‐reported outcomes (PROs) by national health authorities	
**How are PRO perceived by National Health Authorities, both regulatory and health‐technology assessment bodies?** National Health Authorities proactively ask to include PROs data to support new technologies evaluation processes National Health Authorities do not ask proactively about PROs data/do not necessarily require PROs data	2/13 11/13
**Regarding pricing and reimbursement decision‐making processes, are PRO being used?** Yes, they are included within pharmacoeconomics	2/13
**Do you believe PRO data should be included as part of the standard process when evaluating a new product (if not already)?** Yes	12/13

*Note*: Number of participants to the interview who gave any answers out of the total number of participants are provided.

Abbreviation: PRO = patient‐reported outcomes.

All the participants stated that the PRO are collected at their own countries in the context of clinical trials and/or specific projects. In two countries (Austria and Sweden), the PRO are routinely/almost routinely collected during everyday clinical practice also outside clinical trials and/or specific projects and in five countries (Czechia, Denmark, France, Greece, and Portugal), the PRO are collected during everyday clinical practice only at the discretion of the treating physicians. All interviewed people responded that clinicians are involved in the collection and analysis of the PRO, with the additional support of nurses in four (Austria, Denmark, Romania, and Sweden) and psychologists in three (Austria, Czechia, and Greece) countries.

In all seven countries where the PRO are obtained during everyday clinical practice routinely/almost routinely or at the discretion of the treating physicians, they are collected during either the access, treatment, or follow‐up phases of the care of people with epilepsy. Both paper‐based and electronic questionnaires are used to collect data, with a preference for the former in Czechia and for the latter in Austria. In all seven countries, questionnaires to evaluate the impact of the illness or the treatment on the mental health (e.g., anxiety, depression, and cognition) and questionnaires to assess the impact of treatment in terms of safety/tolerability are adopted; in Sweden, questionnaires to evaluate work productivity (e.g., Work Productivity and Activity Impairment Questionnaire) are also used. In Portugal PRO collected also include generic preference‐based PRO, such as the EQ‐5D or the SF‐6D, or generic health profiles, such as the SF‐36. The main uses of the PRO included the support in evaluating the efficacy and/or safety of the treatment and in informing decisions on the general management of people with epilepsy.

Across the different countries, the PRO are taken into account and included in the processes of drug evaluation and selection in Austria, Czechia, Greece, Portugal, and Sweden whereas PRO collection is deemed a priority in Austria, Czechia, France, Portugal, and Sweden. Among the participants, nine would rather dedicate more time and space to the collection of PRO, and the remaining four—from Austria, Czechia, Portugal, and Sweden—believed that the time currently devoted to PRO is already adequate.

There was a complete consensus among the respondents about the favorable impact that the PRO can have in terms of clinical outcomes, healthcare resources utilization, and general patient satisfaction. In the perspective of all participants, the PRO are useful to measure the success of a pharmacological treatment and to orient the choice toward one treatment over another; there was complete unanimity also about the opportunity to include the PRO among the indicators of the value of an intervention. Most of the participants agreed on the role that PRO can have to support specific label claims and product indications, while respondents from Belgium, Greece, and Italy were more skeptical about this issue.

Only participants from Portugal and Sweden answered that the PRO are perceived as very important by the National Health Authorities of their respective countries—which proactively ask to include such data to support the assessment of new technologies—and are considered in pricing and reimbursement decision‐making processes. In contrast to the currently restricted role of PRO in formal drug evaluation processes, almost all the interviewees believed in the importance to include the PRO as part of the standard procedure when evaluating a new product; in this regard, the participant from France highlighted that only long‐term data outcome would be informative, but such information is generally not available when a new drug is undergoing the approval and marketing authorization.

All the interviewees believed that the role of PRO will increase in the future, and all except those from Belgium, Italy, and Slovakia stated that digitalization will support the boost in the use of PRO and their evaluation.

## 4 DISCUSSION

This study depicted a great heterogeneity in the collection and use of the PRO across different European countries.

In Austria and Sweden, the collection, analysis, and use of the PRO take place both in clinical trials and everyday clinical practice. In these countries, they indeed represent a priority in the epilepsy management and have now become part of the standard of care and patient evaluation. In European countries like Czechia, Denmark, France, Greece, and Portugal the PRO are collected in clinical practice at the discretion of the clinician. The PRO are collected during the different phases of patient care, including (I) the patient access to mainly investigate the quality of life, impact of the illness on work productivity, and concerns or expectations from the treatment; (II) the treatment to mainly define the impact of the therapy on quality of life, comorbidities, and mental health (e.g., anxiety, depression, cognitive functioning) as well as functional status; (III) the follow‐up to monitor the patient's health over time. In all these countries, the PRO are thought to capture a more comprehensive view of treatment effectiveness and tolerability, to inform decisions on either the treatment or the general management (e.g., need for a visit), to measure and improve the utilization of health care resources.

Data collection takes place through both paper and electronic questionnaires. In most cases, the collection and analysis of data are entrusted entirely or almost entirely to the clinicians, who may count on the additional support of other health care professionals such as nurses and psychologists in a minority of the centers. Of note, data collected are inserted in National Registries in Sweden, where they can be consulted and updated by all specialists that take care of a patient. The respondents judged the tools as overall valid to obtain meaningful data, but they also highlighted some caveats. The instruments used to evaluate the PRO can be sometimes too simplistic and, hence, not sensitive enough to capture the patient's perspectives in a holistic manner. Importantly, the clinician‐patient dialogue is broadly considered the most effective method for collecting feedback from the patients, as well as the optimal way for discussing and investigating any concerns related to the questionnaires.

In Belgium, Italy, Poland, Romania, Slovakia, and the United Kingdom, the PRO are considered part of the “good clinical practice.” They, however, are not part of the routine assessment of people with epilepsy, but rather are evaluated exclusively, almost exclusively or mostly within the context of clinical trials or specific projects. The lack of time, human and economic resources is the main barriers for the implementation of the PRO in clinical practice. In these countries, the impact of the PRO in guiding the management of patients is, hence, quite marginal.

Despite the geographical differences in the actual use of the PRO in clinical practice, there is a uniform perception throughout the European countries involved in this interview that a more robust inclusion of the perspective of the patient in the management of his/her condition is a fundamental goal. All respondents to the interview believed that the PRO and the evidence obtained from them can improve the patient management and rational use of healthcare resources, can contribute to measure the efficacy of pharmacological and nonpharmacological treatments, and they should be included among the indicators of the value of a treatment, providing additional cues to orient the choice of one treatment over another. The assessment of the frequency of seizures is, indeed, perceived by the interviewees as not sufficient to guarantee the general well‐being of the patient. The current evaluation approach, mainly based on the frequency of seizures as a benchmark to define the success of a treatment, needs to be moved toward a more holistic vision, attributing greater importance to parameters such as the impact of the disease and the intervention on the patient's quality of life. The importance of including the patient in the selection of the PRO is also underlined.

According to the results of the interview, the perception of the PRO by National Health Authorities differs across the countries. In Portugal and Sweden, the National Health Authorities proactively promote the collection and analysis of the PRO, requiring the presence of such data among the information provided in the submission dossiers of new drugs. The PRO are also included in the pricing and reimbursement decision‐making processes, alongside efficacy and tolerability data, recognizing the ability of the PRO to inform about the healthcare costs and economic impact of a treatment. Of note, in Sweden, Health Technology Assessment (HTA) authorities emphasize cost‐effectiveness along with human dignity and the need‐solidarity principle for the prioritization of resources. Swedish HTA agencies see PROs as influential to the understanding of the benefit from the patient perspective, and the use of Quality Adjusted Life Year (QALY) is the used method to understand quality of life. In the remaining European countries, the PRO are administered as part of clinical trials and have a more limited role in supporting the evaluation processes, including those for reimbursement purposes, of new treatments that have not yet received marketing authorization. In these countries, PRO data can be included whenever available, but they are generally not mandatory.

In the United Kingdom, the National Health Service has supported nationwide PRO collection in disease areas outside of neurology by encouraging PRO assessment and performing data linkage to national datasets such as the Hospital Episode Statistics (HES) database for hip and knee replacement (NHS Digital, 2023; Provisional Patient Reported Outcome Measures (PROMs) in England—or Hip & Knee Replacement Procedures 2023). There is no current national PRO initiative for epilepsy in the United Kingdom, and this is thought to be due to a number of reasons including the paucity of current evidence demonstrating the health and economic benefits for routine PRO measurement for epilepsy and concerns about the feasibility and availability of the resources to collect and analyze the data. Of note, the National Health Service in the United Kingdom has recently stopped the data collection of PRO measures for some conditions such as varicose vein surgery due to burden and concerns regarding impact (Letter from NHS England regarding changes to PROMs collection of groin hernia & varicose vein procedures, 2017). While collection of PRO in routine healthcare seems a good thing to do, implementation requires additional resource, systems to do so and time, which all incur healthcare costs. In this regard, so far there is not any robust evidence that doing so is a cost‐effective healthcare intervention.

Importantly, all the specialists interviewed believed that the PRO could offer the opportunity of a more comprehensive and multidimensional assessment of the whole effects of a new treatment on the patients and the health care system, and they should be included as part of the standard process when evaluating a new pharmacological or nonpharmacological intervention.

Among the current unmet needs, the interviewees identified the present lack of standardized methods for collecting PRO in different centers and setting, and the absence of a fully comprehensive dashboard that may be consulted by the physician and provide all the information about the clinical management of the patient, including the PRO. Furthermore, scales and questionnaires for the assessment of the PRO are not always easy‐to‐use, they can be too complicated for the patients and sometimes are not fully adaptable to the individual sociocultural situation, or translation in the national language is not available. Importantly, the patient empowerment is felt to be still limited, and the support from the National Health Authorities as well as the resources invested are not considered adequate.

Some suggestions to improve and promote the collection, analysis and use of the PRO included the implementation of validated and easy‐to‐use standardized tools, the active involvement of patients and their associations in the selection of the PROs, the release of recommendations to guide the use of the PRO in clinical practice, and the production of a Global Economic Assessment to highlight the impact of the PRO on direct and indirect health care costs. Importantly, initiatives to develop core outcome sets and measurement instrument sets for people with epilepsy are ongoing, and the output of this work could be useful in identifying which PRO to routinely capture in different settings, including clinical practice and clinical trials both in children and adults with epilepsy (Chiang et al., 2023; ICHOM, 2023; Mitchell et al., 2022).

Finally, all the interviewees trusted that the collection of the PRO will undergo a positive boost in the future, and digitalization will play an increasing role. Although the actual ways via which digitalization will increase PRO adoption and evaluation in clinical practice were not specifically discussed and detailed, it was generally believed that digitization will facilitate relevant data collection and analysis as well as their integration in clinical decision‐making. For instance, it is anticipated that digitalization may reduce time spent for PRO collection during in‐person consultations and may allow real‐time monitoring of PRO, in the context of telemedicine, thereby enabling improved patient‐physician interactions. In Sweden, there are plans to expand data collection to caregivers too, with the development of “Family Reported Outcomes,” together with the development of specific tools dedicated to the self‐sufficient and nonself‐sufficient elderly patients. In Austria, the development of a tool to assess the general state of people with epilepsy is ongoing, and the support of a nurse dedicated to the collection of the PRO is foreseen in France.

This interview was an explorative but ambitious attempt to provide a map and synthetic overview of the usage and impact of PRO in epilepsy across Europe. Some shortcomings need, however, to be acknowledged. A limited number of experts were interviewed, and they were not selected according to a systematic approach, but rather represented a convenience sampling. Only one expert was interviewed for each country, and only some European countries were represented with some others left out although they have a high impact in the epilepsy care. The respondents to the interview worked at tertiary, academic centers, and their experiences could not be necessarily representative of the national scenarios and could not take into account differences within any respective countries. The different background of the interviewees, namely medical sciences, health economy, and epidemiology, may have represented a source of bias and heterogeneity in depicting the panorama at country level. Since only one individual per country was interviewed, it is challenging to obtain a broader picture of the European clinical practice reality with PRO. In this regard, the inclusion of nontertiary centers or studies conducted by epilepsy societies could provide a more comprehensive view. In addition, critical aspects in PRO among people with epilepsy like evaluating the handling of specific situations such as acute situations or status epilepticus to enhance healthcare resource utilization, or during initial episodes and diagnosis, have not been addressed separately. Finally, the interview was mainly based on closed questions, which allowed to substantially perform a quantitative analysis only; this approach, however, could be advantageous if the project will be extended to a broader number of participants as it would allow to summarize and analyze the results more easily.

In summary, differences exist in attitudes and perspectives about the PRO in epilepsy across different European countries. An active, strategic plan with the involvement of key stakeholders is warranted to harmonize the measurement of the PRO and ensure they can be relevant to people with epilepsy and health services.

## AUTHOR CONTRIBUTIONS

**Kristl Vonck**: Writing‐review and editing; conceptualization; investigation. **Arnaud Biraben**: Writing‐review and editing; investigation. **Magdalena Bosak**: Writing‐review and editing; investigation. **Poul Jørgen Jennum**: Writing‐review and editing; investigation. **Vasilios K Kimiskidis**: Writing‐review and editing; investigation. **Petr Marusic**: Writing‐review and editing; investigation. **James W. Mitchell**: Writing‐review and editing; investigation. **Lara N. Ferreira**: Writing‐review and editing; investigation. **Martina Ondru.ová**: Writing‐review and editing; investigation. **Adrian Pana**: Writing‐review and editing; investigation. **Ulf Persson**: Writing‐review and editing; investigation. **Tim J von Oertzen**: Writing‐review and editing; investigation. **Simona Lattanzi**: Writing‐review and editing; writing‐original draft; conceptualization; investigation; supervision; data curation.

## CONFLICT OF INTEREST STATEMENT

Magdalena Bosak received honoraria for publications from Sanofi; honoraria for lectures, travel expenses and conference fees from Sanofi, Adamed, Angelini Pharma, Neuraxpharm, UCB Pharma, and for the participation in advisory meetings from Sanofi and UCB Pharma outside the submitted work. Vasilios K Kimiskidis received grants from UCB Pharma and personal fees from Arriani and UCB Pharma outside the submitted work. Petr Marusic has received honoraria for lectures from Angelini Pharma, Egis, Eisai, and UCB Pharma, and has served on advisory boards for Angelini, Biogen, and UCB Pharma outside the submitted work. James W. Mitchell reported grants from the Association of British Neurologists and Guarantors of Brain Charity, personal fees from the International Consortium for Health Outcomes Measurement and the UNEEG medical, nonfinancial support from UCB Pharma, American Academy of Neurology, Epilepsy Foundation America, and Angelini Pharma outside the submitted work. Tim J von Oertzen has received payment or honoraria for lectures, presentations, or manuscript writing support from Angelini Pharma, Eisai, GW Pharmaceuticals, Jazz Pharma, Livanova, and UCB Pharma and has served on advisory boards for Angelini Pharma, Arvelle Therapeutics, GW Pharmaceuticals, Jazz Pharma, and Zogenix Pharma outside the submitted work. Simona Lattanzi has received speaker's or consultancy fees from Angelini Pharma, Eisai, GW Pharmaceuticals, Medscape, and UCB Pharma and has served on advisory boards for Angelini Pharma, Arvelle Therapeutics, BIAL, Eisai, GW Pharmaceuticals, and Rapport Therapeutics outside the submitted work. All authors reported personal fees from Ethos S.r.l. in compensation of time for the undertaking of the expert interviews.

### PEER REVIEW

The peer review history for this article is available at https://publons.com/publon/10.1002/brb3.3342.

## Supporting information


**Supplementary appendix**. Structured interview about patient‐reported outcomes.

## Data Availability

Anonymized data will be shared upon reasonable request of any qualified investigator.
