# Supplementary material for: Usage and impact of patient‐reported outcomes in epilepsy
Source: Brain Behav. 2023 Nov 23;13(12):e3342. doi: 10.1002/brb3.3342 (PMC10726862; doi:10.1002/brb3.3342)
Supplement: Supplementary file 1 — Supplementary appendix. Structured interview about patient‐reported outcomes. [file BRB3-13-e3342-s001.docx]

**Appendix. Structured interview about Patient-Reported Outcomes**

**Part I. Patient reported outcomes (PROs): collection during patient access, treatment, and follow-up**

1. Are PRO collected within your center?

☐ Yes

☐ No

1. Which professional figure is in charge of the collection and analysis of PRO in your center?

☐ Clinician

☐ Nurse

☐ Other

1. When PRO are collected?

☐ In clinical trials and/or specific projects

☐ Routinely/almost routinely during everyday clinical practice

☐ At the discretion of the treating physicians during everyday clinical practice

1. If PRO are collected during everyday clinical practice, are they collected in the patient access phase, investigating quality of life, impact of the illness on the productivity, concerns, or expectations from the treatment?

☐ Yes

☐ No

1. If PRO are collected during everyday clinical practice, are they collected during the treatment phase, as parameters to define the impact of the therapy on quality of life and the patient's functional status?

☐ Yes

☐ No

1. If PRO are collected during everyday clinical practice, does the collection continue during the follow-up phase?

☐ Yes

☐ No

1. If PRO are collected during everyday clinical practice, how are PRO data used in your center?

☐ To evaluate the efficacy and/or safety of the treatment

☐ To inform decisions on the treatment

☐ To inform decisions on the general management (e.g., need for a visit or other resources)

☐ To help measure health care resources utilization and improve efficiency

1. If PRO are collected during everyday clinical practice, which tools does your center use to collect them?

☐ paper-based questionnaire

☐ electronic questionnaire

1. Within your center, are PRO considered and included in the drug evaluation and selection process?

☐ Yes

☐ No

1. If you could, would you dedicate more time/space to PRO collection?

☐ Yes

☐ No

1. Is PRO collection considered a priority at your center?

☐ Yes

☐ No

**Part II. Value and impact of PRO in the decision-making process**

1. Do you think that the use of PRO and the evidence obtained from them, can offer a concrete advantage in terms of clinical outcome, rational healthcare resources, and general patient satisfaction?

☐ Yes

☐ No

1. Do you think that PRO collection and evaluation are useful to measure the success of a pharmacological treatment?

☐ Yes

☐ No

1. Do you think that PRO collection and evaluation are useful to improve efficiency in patients’ management?

☐ Yes

☐ No

1. Do you think that PRO can be included among the indicators of the value of a drug?

☐ Yes

☐ No

1. Do you believe that PRO are useful indicators in orienting the choice towards one drug over another?

☐ Yes

☐ No

1. Do you believe that PRO should be used to support label claims (product indications)?

☐ Yes

☐ No

**Part III. Interest for and use of PRO by national health authorities**

1. How are PRO perceived by National Health Authorities, both regulatory and health-technology assessment bodies?

☐ PRO are very important outcomes, National Health Authorities proactively ask to include such data to support new technologies evaluation processes

☐ National Health Authorities do not consider PRO crucial/do not necessarily require PRO data

1. Regarding pricing and reimbursement decision-making processes, are PRO being used?

☐ Yes, they are included within pharmacoeconomics (e.g., Building Information Modeling, cost-effective analysis)

☐ No

1. Do you believe PRO data should be included as part of the standard process when evaluating a new product (if not already)?

☐ Yes

☐ No

1. What do you think will be the future direction on PRO?
